# Macroalgal-Associated Dinoflagellates Belonging to the Genus *Symbiodinium* in Caribbean Reefs

**DOI:** 10.1371/journal.pone.0002160

**Published:** 2008-05-14

**Authors:** Isabel Porto, Camila Granados, Juan C. Restrepo, Juan A. Sánchez

**Affiliations:** Departamento de Ciencias Biológicas-Facultad de Ciencias, Laboratorio de Biología Molecular Marina (BIOMMAR), Universidad de los Andes, Bogotá, Distrito Capital (DC), Colombia; University of Sheffield, United Kingdom

## Abstract

Coral-algal symbiosis has been a subject of great attention during the last two decades in response to global coral reef decline. However, the occurrence and dispersion of free-living dinoflagellates belonging to the genus *Symbiodinium* are less documented. Here ecological and molecular evidence is presented demonstrating the existence of demersal free-living *Symbiodinium* populations in Caribbean reefs and the possible role of the stoplight parrotfish (*Sparisoma viride*) as *Symbiodinium* spp. dispersers. Communities of free-living *Symbiodinium* were found within macroalgal beds consisting of *Halimeda* spp., *Lobophora variegata*, *Amphiroa* spp., *Caulerpa* spp. and *Dictyota* spp. Viable Symbiodinium spp. cells were isolated and cultured from macroalgal beds and *S. viride* feces. Further identification of *Symbiodinium* spp. type was determined by length variation in the Internal Transcribed Spacer 2 (ITS2, nuclear rDNA) and length variation in domain V of the chloroplast large subunit ribosomal DNA (cp23S-rDNA). Determination of free-living *Symbiodinium* and mechanisms of dispersal is important in understanding the life cycle of *Symbiodinium* spp.

## Introduction

Coral reefs maintain an incredible level of productivity considering the oligotrophic waters in which they are found. The high productivity and rapid growth exhibited by reef-building corals can be attributed to their association with symbiotic dinoflagellates referred to as zooxanthellae [1]. Other symbiotic reef dwellers include platyelminths, mollusks, sponges and protists [2], [3]. There is an increasing understanding of *Symbiodinium* outside their hosts, documented from temperate waters of New Zealand [4], benthic sands in Hawaii [5], water column in China [6], and in coral reef sediments [7].

The complete life stages and sexual reproduction of most dinoflagellates, including *Symbiodinium* spp., are not well understood [8]. *Symbiodinium* spp. within the host exhibits asexual reproduction through mitosis. However, genetic evidence including no linkage disequilibrium and high allelic diversity suggest the presence of recombination and sexual reproduction [9], [10]. Furthermore, Gonyaulacales and Gymnodiniales, Suessiales (*Symbiodinium*) sister groups, exhibit nuclear cyclosis and meiosis according to nuclear and mitochondrial DNA [11]–[13].

Characterization of free-living *Symbiodinium* spp. is important for understanding coral reefs and the coral-symbiont dynamics [14]. There are two modes of zooxanthellae acquisition, vertical (from the parental colony to the eggs) and horizontal (uptake from the environment by aposimbiotic eggs or larvae) [15]. It is important to know if there exists an environmental *Symbiodinium spp.* reservoir from where corals with horizontal symbionts acquisition mode will obtain *Symbiodinium* spp.

The second part of this study addresses dispersion exhibited by *Symbiodinium* spp. carried on by *Sparisoma viride*. This is especially interesting considering their limited motility capacities [16]. Dispersion mechanisms should include oceanic currents, nearshore currents, and/or some living disperser, such as reef fishes. There is compelling evidence showing corallivorous fish have the ability to disperse viable zooxanthellae capable of establishing symbioses with anemones [17]. If free-living *Symbiodinium* spp. live in other habitats such as macroalgal beds and turfs, herbivorous fishes should serve as a dispersing mechanism, disposing partially digested and viable free-living *Symbiodinium* in various habitats.

*Sparisoma viride*, known as the stoplight parrotfish, belongs to a functional group of herbivorous that has a significant effect in Caribbean reefs due to their bioerosion, sediment removal and foraging behaviors [18], [19]. In addition, this fish is considered to have a big effect on corals, inflicting numerous injuries directly to coral colonies [20]. Bruggemann [21] found that *S. viride* feeds exclusively on algae; 95% of the bites registered were done on algae associated with dead coral, while only a 3.6% was done to live coral (“spot biting”), presumably a territorial behavior. It was noted that occasionally the fish spite out the food, particularly when they ingested live corals [21]–[23].

Coral reef scientists have started to appreciate the important role of parrotfishes as ecosystem engineers and keystone species, leading to cascade effects and positive feedbacks directly to coral reef health and resilience. Parrotfishes have record rates of bioerosion in some coral reef areas and their coral grazing effect promotes reef-building coral diversity [18]. Marine protected areas effectiveness, nowadays under the threats of global warming and coral bleaching, depends greatly on ecosystem resilience, where the grazing effect of parrotfishes, as well as other herbivores, is the key role to maintain the functional dominance of reef builders such as corals and coralline algae [24], [25].

The aims of this paper were (i) to present both ecological and molecular evidence demonstrating the existence of macroalgal-associated demersal free-living *Symbiodinium* spp. populations in coral reefs and (ii) to examine the potential role of reef fishes (*Sparisoma viride*) as free-living *Symbiodinium* spp. dispersers.

## Materials and Methods

### Field Collections

**Figure 1:**
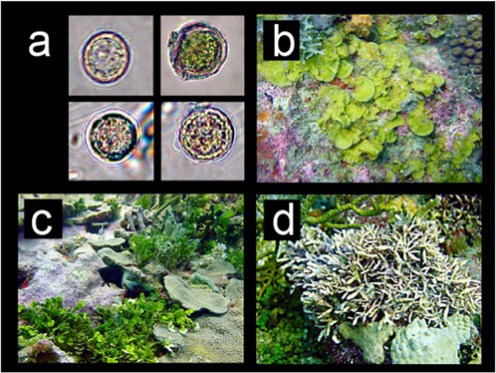
Photographs of free-living *Symbiodinium* habitats and individuals. A. Photographs of *Symbiodinium* sp.-cell like sampled directly form macroalgal beds (Tobago samples). B–D. Bed-forming macroalgae samples where free-living zooxanthellae were sampled. B. *Lobophora variegata*. C. *Halimeda* spp. D. *Amphiroa tribulus*. (B–D. Salmedina Banks, 15 m, Cartagena, Colombia).

Samples were collected by means of SCUBA diving from Cartagena, Santa Marta, and Tobago (Trinidad and Tobago) reefs, between 2005 and 2006. Sites (reefs), depths, and number of samples are summarized in the Table 1. Sampling was conducted by applying gentle pressure on several algal beds (mainly of *Halimeda* spp., *Dictyota* spp., *Lobophora variegata*, *Caulerpa* spp., and *Amphiroa* spp.) that where next to coral colonies, and withdrawing particles that were in suspension with a 60 ml syringe (Figure 1). Water above the algal beds were sampled prior to agitation, these samples served as experimental controls. One set of water samples with the suspended particles was transferred to eppendorf tubes containing DMSO or 96% ethanol for molecular analysis. The other set of samples was cultured quickly after collection. The collection of *Symbiodinium* spp. from the feces of *Sparisoma viride* was done by means of SCUBA diving. Using 100 ml sterile tubes, feces from the water immediately after being released from the fish were collected and kept in sterile bags. Experimental controls from the water column were sampled. The collected samples were then cultured at the laboratory.

**Table 1:** Summary of the sampling scheme. Reefs (sites), depths and number of samples (environmental and *Sparisoma viride*) collected.

Locality	Site	Depth (m)	# of samples (experimental controls)
Cartagena-Colombia	La despensa	22	21(2)
	Pavitos	14	9
	Tesoro island fore-reef terrace	14	21
	Periquitos	0–3	9
	Periquitos	10	5
	Periquitos	30	2
Santa Marta-Colombia	Paso del Ángel	20	40 (12)
	Salidero	20	40 (8)
Trinidad y Tobago	South of Corvo point	20	9
	Booby Island	3	6(3)

### Cultures

F/2 medium [26], shown to be a suitable medium for culturing *Symbiodinium* spp. [27], [28], was prepared using seawater passed through 0.45 micrometers filter paper. In addition, the antibiotic chloranphenicol was used (1 mg ml^−1^) to avoid bacteria contamination. The cultures were started in sterilized test tubes, sealed with cotton, and exposed to a photoperiod 12 hrs light: 12 darkness at 27–30°C. Direct observations under microscope of the cultures were carried out to verify zooxanthellae presence. When zooxanthellae growth was noticed, a sample was taken out and DNA extracted.

### DNA Extraction and Molecular Analyses

DNA from cultured cells and environmental samples were extracted using the CTAB phenol-chloroform-isoamyl alcohol protocol [29]. Two gene regions, ITS2 rDNA and chloroplast 23S ribosomal DNA (rDNA) domain V (cp23S) were used to detect *Symbiodinium* spp. using polymerase chain reaction (PCR). The ITS2 region was amplified using the forward primer “ITSintfor2” (5′GAATTGCAGA ACTCCGTG-3′) and a modified reverse primer ‘ITS2CLAMP (5′CGCCCGCCGC GCCCCGCGCC CGTCCCGCCG CCCCCGCCC GGGATCCATA TGCTTAAGTT CAGCGGGT-3′) [30], with a GC clamp (underlined). PCR reactions were carried out using LaJeunesse (personal communication) “Touchdown” protocol, in a MyCycler thermocycler (BioRad) under the following conditions: an initial denature period at 92° for 3 min, followed by 35 cycles of 30 sec at 92°C, 40 sec at 48°C and 30 sec at 72°C and a final extension period of 10 min at 72°C. Primers used to amplify cp23S were 23SHYPERUP (5′-TCAGTACAAATAATATGCTG-3′) and 23SHYPERDNM13 (5′-GATAACAATTTCACACAGGTTATCGCCCCAATTAAACAGT-3′) [28]. The PCR conditions were: an initial denature period of 3 min at 94°C, followed by 40 cycles of 1 min at 94°C, 30 s at 54.2°C, 30 s at 72°C and a final extension period of 5 min at 72°C [31]. The ITS2 products were loaded in a Denature Gradient Gel Electrphoresis (DGGE) containing a gradient of 3.15 M urea/18% deionized formamide to 5.6 M urea/37% deionized formamide; bands were excised from the gels, reamplified and sequenced. The cp23S rDNA were electrophoresed in polyacrylamyde gels. As in DGGE, the bands were excised from the gels, reamplified and sequenced.

To determine the identity of the ITS2 and cp23S sequences, phylogenetic trees were generated using sequences from clades A, B and C from the Caribbean. These sequences were taken from the Santos lab (Auburn University) webpage database and from *Symbiodinium* spp. isolated from corals of the same reefs that the water and fecal samples were collected (unpublished) (Figure 1B). Phylogenetic trees were obtained using MrBayes [32] and maximum likelihood-ML (PAUP*) using the best-fit model and parameters according to Modeltest [33] and the Akaike Information Criterion.

## Results

Bed-forming macroalgae, such as *Halimeda* spp., *Lobophora variegata, Amphiroa* spp., and *Caulerpa prolifera* (figure 1B–D), were the most frequent non-coral habitats for free-living *Symbiodinium* spp. in coastal Colombia. *Dictyota* spp. was the most prevalent in Tobago (Table 2). Near bottom water column controls were negative for all Cartagena samples but were positive in the Tobago (Booby Island) experimental controls (30%). We also found occasional positive amplifications in sediments around corals (3%), like previous works conducted by Coffroth [7] show.

**Table 2:** Summary of the free-living *Symbiodinium* spp. clades found in the sampled reefs, depths, and substrates.

Location	Depth (m)	Free-living zooxanthellae Clade	Substrate
Cartagena (Isla Grande, La despensa)	22	C	Sediments around corals
Cartagena (Pavitos island)	14	B184, C	*Halimeda* spp. (including *H. opuntia*)
		A4, C, B184	*Halimeda* spp. (including *H. opuntia*)
		B184, C	*Lobophora variegata*
Cartagena (Tesoro island fore-reef terrace)	14	B184, C	*Lobophora variegata*
		B184, C	*Lobophora variegata, Amphiroa sp.*
		A4, C, B184	*Amphiroa* spp.
		A4, C, B184	Sediments around corals
Cartagena (Barú: Periquitos island)	0–3	B184	*Halimeda* spp., (including *H. opuntia*)
Tobago (South of Corvo point)	18	B184	*Dictyota* sp.

**Figure 2:**
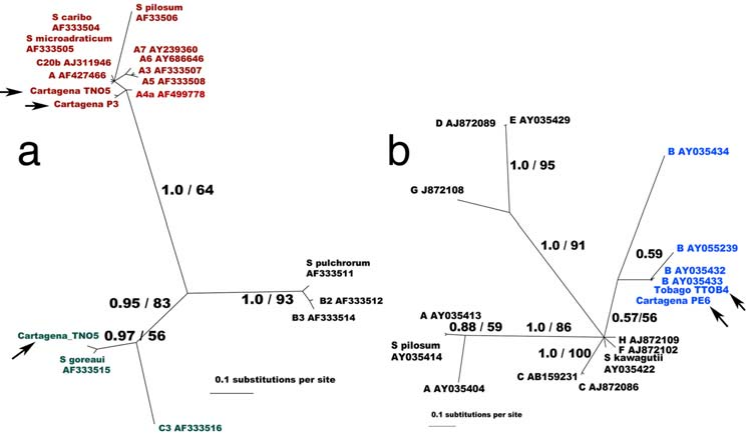
Unrooted star phylograms of *Symbiodinium* spp. Topologies were obtained with Bayesian inference (support for major clades are Bayesian clade credibility/maximum likelihood 100 bootstrap replicates). A. ITS2 phylogenetic hypothesis. B. cp 23S phylogenetic hypothesis. Terminal branch names correspond to the zooxanthellae clade letter plus GenBank accession numbers except for new free-living sequences.

Positive molecular identifications of free-living *Symbiodinium* spp. associated with macroalgal beds were found both in environmental samples (table 2) and in cultures. *Symbiodinium* spp.-like cells were observed directly under the microscope further verifying this association (Figure 1A). Overall, PCR amplifications of DNA isolated from samples collected at different locations and several habitat/depth replicates, yielded *Symbiodinium* spp. products from the planktonic demersal genomic DNA (Tables 2 and 3). Of the 9 sampled reefs, we detected *Symbiodinium* spp. in 7 reefs in several replicates, having 53% of positive PCR products from the non-cultured samples (table 3). Sequencing (ITS2 accession numbers EU139607, EU139608 and cp23S accession numbers EU139605, EU139606) and comparative analyses with known sequences from cp23S and ITS2 corroborated the identity of the most frequent PCR amplification products belonging to *Symbiodinium* spp. clades A (*Symbiodinium* [ = *Gymniodinium*] *linuchae*, A4: ITS2), B (B184; cp 23S) and C (ITS2) (Figure 2A–B; Table 2). Those *Symbiodinium* spp. types are also present in symbiosis with cnidarian host species, such as *Porites astreoides* (A4) and *Pseudopterogorgia acerosa* (B184). The identity of isolated type of clade C is not precise, due to the large polytomy present in this group.

**Table 3:** Summary of the cultures of free-living zooxanthellae and zooxanthellae isolated from the feces of *Sparisoma viride*.

Substrates	Successful Cultures	Non-successful Cultures
*Caulerpa* spp.	5	3
*Halimeda* spp.	8	0
*Dictyota* spp.	4	2
*Amphiroa* spp.	7	1
*Lobophora* spp.	10	0
Total (Percentage of success from bed-forming macroalgae)	34 (85%)	6
Feces from *S. viride* (Percentage of success from *S. viride*)	26 (65%)	14

Free-living *Symbiodinium* spp. were successfully cultured (Table 3). The cultures of *Symbiodinium* spp. cells from the feces of *S. viride* were also successful although a few experimental controls from the water column presented a positive band in the molecular analysis. Positive DNA extractions from commercial cultures (Carolina, USA) of other dinoflagellates such as *Amphidinium carterae* and *Gymnodinium* spp., as well as a diatom consortium, were always negative for the targeted cp23S DNA regions, reassuring on the zooxanthellae specificity of the molecular method used.

## Discussion

This study found *Symbiodinium* spp. cells in demersal plankton habitats [34], [35] in the coral reef areas of coastal Colombia (Cartagena and Santa Marta), Trinidad and Tobago (Charlotteville, Tobago). Water samples collected directly from macroalgal beds and parrotfish feces were both found to contain free-living *Symbiodinium* spp. Positive identification was achieved by molecular analysis and viability by culturing of *Symbiodium* spp. (Tables 2–3).

Macroalgae, with their intricate branching networks, have high surface to volume ratio, providing substrate, light attenuation, and refuge (Figure 1B–D) and may be a major habitat for free-living *Symbiodinium* spp. on Caribbean coral reefs. It is important to mention that there is still uncertainty whether *Symbiodinium* spp. live as epiphytic dinoflagellates such as *Gambierdiscus toxicus* [36] or move freely inside the interstitial spaces within algae. Nonetheless, these findings are in accordance with the low motility and buoyancy of *Symbiodinium* spp. [35], [37] as demersal organisms and with the occurrence of *Symbiodinium* spp. in gut contents of macroalgal-feeders such as the Queen conch *Strombus gigas* [30] and coralliivorous fish [17].

The occurrence of *Symbiodinium* spp. in other habitats helps to explain the large diversity that this genus has shown. It has been proposed that niche diversification of *Symbiodinium* spp. outside their cnidarian host could have maintained symbiont diversity through ecological shifts [30]. Zooxanthellae types A4 and B184 are two common symbionts of scleractinian and soft corals in the study area (unpublished), as well as other sampled Caribbean locations [38]. Despite the lack of precise type identification, the clade C symbiont is related to other types found in hard corals of the genus *Agaricia* spp. and *Montastraea* spp., both prevalent in the Caribbean reefs. The types found here are a small proportion of the high *Symbiodinium* spp. diversity that can be associated to reef dwellers [30], [39].

In this study, the sampled macroalgal beds were directly adjacent to symbiotic corals. Despite the limited motility capacities of *Symbiodinium* spp., short distance dispersal of free-living *Symbiodinium* spp. from the macroalgal areas to the corals could be achieved by their diurnal swimming behavior or localized hydrodynamic conditions. Given that *S. viride* feces carry viable *Symbiodinium* spp. cells, dispersion on a larger scale could be carried out by coralivores fish. Fecal analysis of other coral predators like *Arothron meleagris*, *Chaetodon auriga*, *Chaetodon unimaculatus* and one nudibranch *Berghia major* from Hawaii have also been shown to contain viable *Symbiodinum* spp. cells. [17].

There is evidence of zooxanthellae uptake from the environment in octocorals (*Briareum asbestinum*) as adults and recruits [7], [14], as well as, in the medusae (*Cassiopea xamachana*) [15] and in scleractinian (*Acropora longicyathus*)[40]. However, there is evidence that the changes of *Symbiodinium* types inside the host are due to shifts in the densities of preexisting types and not from the acquisition of environmental “novel” types [41], [42]. Lastly, there is evidence of a stable symbiotic association over time [43], [44], where there is no environmental *Symbiodinium* spp. uptake.

The term holobiont has been used to describe the tight symbiotic relationship between coral and algae. The reported advantages of the symbiosis are mainly for the coral [45]–[47] and the consequence of the disruption of this nearly obligate mutualism (e.g., coral bleaching) is catastrophic for corals. The advantages for *Symbiodinium* spp. are obtaining inorganic nutrients from host metabolism, including ammonium and phosphate [48], as well as, protection from predation and irradiation (UV). According to the findings in this work, the symbiosis may be facultative for some *Symbiodinium* spp. because they can find some of the benefits that they obtain from the coral in other habitats such as macroalgal beds.

Characterization of free-living *Symbiodinium* spp. populations is also important for understanding the life history of *Symbiodinium* spp. As mentioned above sexual reproduction in *Symbiodinium* spp. is not well understood [10]. However, molecular evidence suggests the presence of recombination and sexual reproduction [11]. It has been further suggested that this mode of reproduction might occur in the free-living stage [2], where the cells may encounter different genotypes.

From this characterization study, we can conclude that there are macroalgal-associated demersal free-living *Symbiodinium* spp. populations in coral reefs and that *Sparisoma viride* feces carry viable *Symbiodinium* cells. This information builds upon our limited understanding of the distribution and dispersion of demersal free-living *Symbiodinium* spp. More research on *Symbiodinium* life history and ecology is urgently needed.
